# Ru–W Pair Sites Enabling the Ensemble Catalysis for Efficient Hydrogen Evolution

**DOI:** 10.1002/advs.202303110

**Published:** 2023-07-12

**Authors:** Weilong Ma, Xiaoyu Yang, Dingding Li, Ruixin Xu, Liangpeng Nie, Baoping Zhang, Yi Wang, Shuang Wang, Gang Wang, Jinxiang Diao, Lirong Zheng, Jinbo Bai, Kunyue Leng, Xiaolin Li, Yunteng Qu

**Affiliations:** ^1^ International Collaborative Center on Photoelectric Technology and Nano Functional Materials Institute of Photonics and Photon‐Technology Northwest University Xi'an Shaanxi 710069 China; ^2^ Oncology Department National Clinical Research Center for Geriatric Disorders Xiangya Hospital Central South University Changsha 410083 China; ^3^ Aeronautical Polytechnic Institute Xi'an 710089 China; ^4^ Beijing Synchrotron Radiation Facility Institute of High Energy Physics, Chinese Academy of Sciences Beijing 100039 China; ^5^ Université Paris‐Saclay CentraleSupélec ENS Paris‐Saclay CNRS LMPS‐Laboratoire de Mécanique Paris‐Saclay 8–10 rue Joliot‐Curie Gif‐sur‐Yvette 91190 France; ^6^ Institute of Intelligent Manufacturing Technology Shenzhen Polytechnic Shenzhen 518055 China

**Keywords:** efficient hydrogen evolution, optimizing elementary steps, Ru single‐atom, Ru–W pair sites

## Abstract

Simultaneously optimizing elementary steps, such as water dissociation, hydroxyl transferring, and hydrogen combination, is crucial yet challenging for achieving efficient hydrogen evolution reaction (HER) in alkaline media. Herein, Ru single atom‐doped WO_2_ nanoparticles with atomically dispersed Ru–W pair sites (Ru–W/WO_2_‐800) are developed using a crystalline lattice‐confined strategy, aiming to gain efficient alkaline HER. It is found that Ru–W/WO_2_‐800 exhibits remarkable HER activity, characterized by a low overpotential (11 mV at 10 mA cm^−2^), notable mass activity (5863 mA mg^−1^ Ru at 50 mV), and robust stability (500 h at 250 mA cm^−2^). The highly efficient activity of Ru–W/WO_2_‐800 is attributed to the synergistic effect of Ru–W sites through ensemble catalysis. Specifically, the W sites expedite rapid hydroxyl transferring and water dissociation, while the Ru sites accelerate the hydrogen combination process, synergistically facilitating the HER activity. This study opens a promising pathway for tailoring the coordination environment of atomic‐scale catalysts to achieve efficient electro‐catalysis.

## Graphical abstract

Atomically dispersed Ru–W pair site doping WO_2_ are fabricated as an alternative to Pt‐based materials for developing efficient alkaline hydrogen evolution reaction catalysts. The synergistic effect of Ru–W sites triggers ensemble catalysis, resulting in superior performance for electrochemical hydrogen generation.

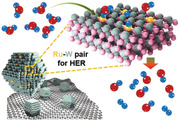

## 1 Introduction

Water electrolysis powered by sustainable electricity offers a cost‐effective and readily available approach for producing green hydrogen and is considered as a key element in achieving future carbon neutrality.^[^ ^1^, ^2^, ^3^ ^]^ The hydrogen evolution reaction (HER) plays a pivotal role in water electrolysis, but the use of noble‐based anode catalysts and expensive proton exchange membranes in acidic electrolytes has led to a quest for efficient alkaline HER electro‐catalysts.^[^ ^4^, ^5^ ^]^ Platinum (Pt) based materials have recently been considered as the benchmark and most capable electro‐catalysts for HER, but their scarcity poses significant limitations for practical applications,^[^ ^6^ ^]^ Ruthenium (Ru), as one of the platinum‐group metals, is being recognized for its potential in developing efficient HER catalysts.^[^ ^7^ ^]^ However, the strong interaction of hydrogen and hydroxyl species over Ru species hinders further water dissociation, hydrogen desorption, and combination processes, resulting in unsatisfactory HER performance.^[^ ^8^ ^]^ Therefore, there is a pressing need to selectively tailor the electronic structure of Ru to fine‐tune the adsorption and desorption abilities of hydrogen and hydroxyl species in a controlled manner, but still challenging.

Single‐atom catalysts (SACs) with tunable local coordination environment represent a promising material platform for modulating the electronic structures of active sites,^[^ ^9^, ^10^ ^]^ as demonstrated by their ability to optimize the free energy of intermediates during the HER process.^[^ ^11^ ^]^ Currently, various Ru SACs have been developed by modulating anions ligands in the first coordination shell, leading to excellent alkaline HER activity, such as Ru SAs@PN,^[^ ^12^ ^]^ Ru‐MoS_2_/CC,^[^ ^13^ ^]^ R‐NiRu,^[^ ^14^ ^]^ RuSA‐Ti_3_C_2_T_x_,^[^ ^15^ ^]^ etc. In these cases, the anions ligands surrounding the Ru site regulate the strong adsorption of hydrogen on Ru, promoting H desorption and combination. However, these isolated Ru sites still exhibit strong hydroxyl adsorption,^[^ ^8^, ^16^ ^]^ as the single active sites are unable to break the scaling relation.^[^ ^17^, ^18^ ^]^ Recently, tailoring the coordination fields through cation regulation has been shown to efficiently improve catalytic activity, particularly for complex multistep reactions.^[^ ^19^, ^20^ ^]^ Electro‐catalysts containing paired metal sites offer significant potential for precisely designing the active moiety to regulate the binding energy of multiple intermediates simultaneously,^[^ ^21^, ^22^ ^]^ which is advantageous for alkaline HER but has received limited investigation thus far.

Inspired by the pioneering works that utilize W as a regulator for Ru nanoparticles,^[^ ^23^, ^24^, ^25^ ^]^ we employ a lattice‐confined strategy to construct Ru–W/WO_2_‐800 catalysts with well‐defined Ru–W pair sites through cation replacement. X‐ray adsorption fine spectroscopy (XAFS) and scanning transmission electron microscope (STEM) analyses confirm that Ru atoms are embedded into the crystalline lattice of WO_2_ and strongly bonded with adjacent W atoms. The optimized catalyst exhibits remarkable activity for HER in alkaline media, with a low overpotential of only 11 mV at 10 mA cm^−2^, a high mass activity of 5863 mA mg^−1^Ru at 50 mV, and robust stability up to 500 h at 250 mA cm^−2^. Operando electrochemical Raman measurements, combined with theoretical calculation, unveil that the superior activity of Ru–W/WO_2_‐800 derives from the synergistic effect of Ru–W pair sites through ensemble catalysis. Specifically, the W sites facilitate rapid hydroxyl transfer and water dissociation, while the Ru sites accelerate the hydrogen combination process, synergistically enhancing the HER activity.

## 2 Results and Discussion

### 2.1 Ru–W Pair Catalysts Synthesis

**Figure 1:**
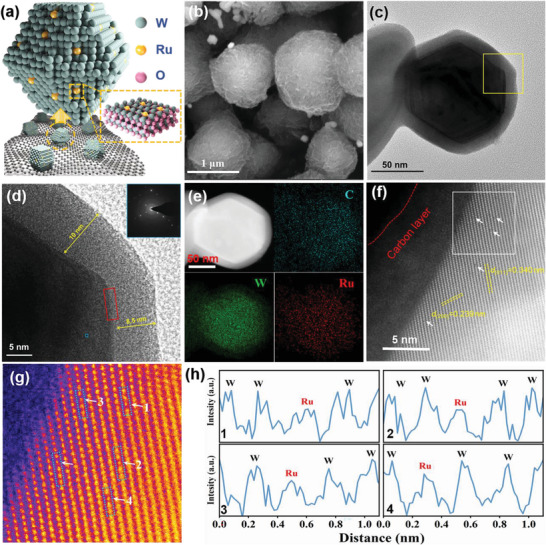
a) Simulative structure and b) SEM image of Ru–W/WO_2_‐800. c,d) TEM images, and e) EDS element mapping of a well‐crystallized WO_2_ in Ru–W/WO_2_‐800. f,g) HAADF‐STEM images of the atom arrays of the WO_2_ crystal and the surface Ru–W atom pairs. h) Line intensity profiles along the (011) plane in (g).

Figure S1 (Supporting Information) illustrates the synthetic process of the Ru–W pair doped WO_2_. Typically, dopamine hydrochloride is first mixed with RuCl_3_ in an acidic solution, followed by the addition of Na_2_WO_4_ to obtain spherical polydopamine‐based metal–organic compounds (Figures S2, S3, Supporting Information). After annealing in an inert atmosphere at 800 °C, Ru–W/WO_2_‐800 with Ru–W dual sites on tungsten oxide is obtained (**Figure** **1**a). To investigate the pivotal role of the Ru–W pair in Ru–W/WO_2_‐800, single atom Ru supported on WO_2_ (Ru_1_/WO_2_‐800) is prepared for comparison (Figure S4, Supporting Information). Additionally, nitrogen‐coordinated single atom Ru (Ru SACs, Figure S5, Supporting Information), commercial Ru/C, commercial Pt/C, and tungsten oxide (WO_2_‐800, Figure S6, Supporting Information) are also used as references.

### 2.2 Physical Characterization

The element mapping results of Ru–W/WO_2_‐800 indicate the existence and the uniform distribution of W and Ru on carbon substrate (Figure S7, Supporting Information). The corresponding W and Ru contents are determined to be 25 and 1.3 wt.% by ICP‐OES (Table S1, Supporting Information), respectively. Figure 1b displays the scanning electron microscope (SEM) images of Ru–W/WO_2_‐800, which exhibits a spherical morphology consisting of an aggregation of nanoflakes. The transmission electron microscope (TEM) image further confirms the random dispersion of WO_2_ particles and nanoclusters within the agminated flakes (Figure S8, Supporting Information). Considering the limited HER activity of single atom Ru or W‐doped carbon,^[^ ^26^, ^27^ ^]^ the potential active sites for HER primarily concentrate on the well‐crystallized WO_2_ particles in Ru–W/WO_2_‐800. The hexagonal morphology of WO_2_ crystals is evident in Figure 1c, with a carbon layer of ≈8–10 nm enveloping the WO_2_ (Figure 1d), which may contribute to its stability. The EDS element mapping confirms the carbon enveloping and the uniform distribution of Ru on WO_2_ particle Figure 1e). The aberration‐corrected high‐angle annular dark‐field‐scanning transmission electron microscope (HAADF‐STEM) is employed to study the surface structure of the WO_2_ particle. As shown in Figure 1f, W atomic arrays with a lattice spacing of 0.239 and 0.340 nm are clearly observed, which correspond to the (200) and (011) plane of the monoclinic WO_2_. Moreover, some dark atomic columns are observed inserting in the W atomic array randomly, which can be distinguished as Ru atoms on the surface of WO_2_ (Figure 1g). Line profiles of the HAADF‐STEM are taken around the Ru atom along the (011) plane, the variable atomic column intensity in Figure 1h demonstrates the replacement of lattice W atom by Ru atom. Moreover, the doping of Ru sites in the WO_2_ lattice is also observed on the WO_2_ clusters in Ru–W/WO_2_‐800 (Figure S9, Supporting Information). For Ru_1_/WO_2_‐800, the HAADF‐STEM image shows the WO_2_ atom array, dark atomic columns of Ru, and the distortion of the atom array around the Ru atom (Figure S10, Supporting Information). However, further investigation is required to distinguish whether the Ru in Ru_1_/WO_2_‐800 and Ru–W/WO_2_‐800 exists as single atoms or as Ru–W coordination through spectral analysis.

**Figure 2:**
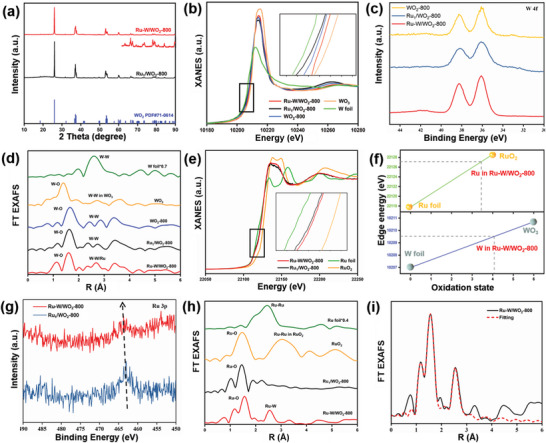
The coordination environment of Ru–W/WO_2_‐800 and Ru_1_/WO_2_‐800. a) XRD patterns. b) W *L_3_‐*edge XANES spectra. c) W 4f XPS spectra. d) W *L_3_‐*edge edge FT EXAFS spectra. e) Ru *K*‐edge XANES spectra. f) The simulative oxidation state of Ru and W in Ru–W/WO_2_‐800. g) Ru 3p XPS spectra. h) Ru *K*‐edge FT EXAFS spectra. i) FT EXAFS fitting curve of Ru–W/WO_2_‐800 at R space.

**Figure** **2**a shows the X‐ray diffraction (XRD) pattern of Ru‐doped WO_2_ catalysts. Both Ru–W/WO_2_‐800 and Ru_1_/WO_2_‐800 display the diffraction peaks belonging to (011), (200), (−202), (−220), and (310) planes of monoclinic WO_2_ at 25.9^o^, 36.7^o^, 37.5^o^, 53.3^o^ and 60.0^o^, respectively, and the peaks of crystalline Ru is absent. To further distinguish the Ru and W combination in Ru–W/WO_2_‐800 and Ru_1_/WO_2_‐800, the XAFS is employed. The oxidation state of W in Ru–W/WO_2_‐800, Ru_1_/WO_2_‐800, and WO_2_‐800 are confirmed similar based on the W L_3_‐edge XANES spectra (Figure 2b), which approximate to +4, consistent with the W 4f X‐ray photoelectron spectroscopy (XPS) spectra (Figure 2c).^[^ ^28^ ^]^ The W L_3_‐edge FT EXAFS spectra are shown in Figure 2d, the differences in the secondary coordination between Ru–W/WO_2_‐800, Ru_1_/WO_2_‐800, and WO_2_‐800 suggest the disturbance of W–W coordination in Ru–W/WO_2_‐800, which may induced by the Ru inserting. To further reveal the interaction of Ru and W in Ru–W/WO_2_‐800, the Ru K‐edge XAFS are performed using Ru_1_/WO_2_‐800, Ru foil, and RuO_2_ as references. Figure 2e shows the Ru K‐edge XANES. The location of the white‐line intensity of Ru–W/WO_2_‐800, which falls between that of Ru foil and RuO_2_, suggests an oxidation state of Ru in Ru–W/WO_2_‐800 between 0 and +4. The linear simulation of edge energy and oxidation state is shown in Figure 2f. The fitting result discloses that the average oxidation state of Ru in Ru–W/WO_2_‐800 is +3.4, slightly higher than the +3.3 in Ru_1_/WO_2_‐800 (Figure S11, Supporting Information), in line with the Ru 3p XPS spectra (Figure 2g).^[^ ^29^ ^]^ The Ru K‐edge FT EXAFAS of Ru–W/WO_2_‐800 displays a dominant peak at ≈1.5 Å (Figure 2h), corresponding to the Ru–O coordination. Moreover, the secondary peak at ≈2.5 Å demonstrates the presence of Ru–W coordination, which is larger than the Ru—Ru bonding in Ru foil (≈2.4 Å) due to the relatively bigger atomic diameter of W. The Ru K‐edge EXAFS fitting results of Ru–W/WO_2_‐800 are presented in Figure 2i and Table S2 (Supporting Information), indicating a coordination number of 3.1 for Ru—O bonding and 0.9 for Ru—W bonding. This confirms the formation of Ru–W pair sites embedded in the lattice of WO_2_ crystalline. For Ru_1_/WO_2_‐800, only a dominant peak at ≈1.5 Å is observed in the Ru K‐edge EXAFS, the absence of Ru–Ru and Ru–W coordination confirms the Ru single atom are absorbed onto the surface of WO_2_.

### 2.3 Electrocatalytic Performance Evaluation

**Figure 3:**
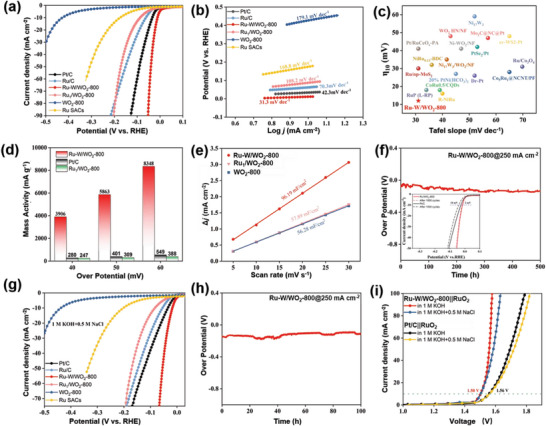
HER evaluation in alkaline medium. a) LSV curves and b) Tafel plots of Ru–W/WO_2_‐800 and reference catalysts. c) Comparison of the HER performance between Ru–W/WO_2_‐800 and other reported catalysts. d) Mass activity of Ru–W/WO_2_‐800, Ru_1_/WO_2_‐800 and Pt/C. e) The current density differences versus scan rate and the corresponding yielded C_dl_ for Ru–W/WO_2_‐800, Ru_1_/WO_2_‐800, and WO_2_‐800. f) Chronopotentiometry test at 250 mA cm^−2^ and the LSV curve of Ru–W/WO_2_‐800 before and after 1000 cycles (insert) in 1 m KOH. g) LSV curves in alkaline simulated seawater of 1 m KOH and 0.5 m NaCl. h) Chronopotentiometry test at 250 mA cm^−2^ of Ru–W/WO_2_‐800 in alkaline simulated seawater. i) Overall water‐splitting performance of the Ru–W/WO_2_‐800||RuO_2_ and Pt/C||RuO_2_ electrode couples.

The synergistic effect of the Ru—W bonding is expected to benefit the HER process. Thus, we employ a three‐electrode system to evaluate the HER performance of Ru–W/WO_2_‐800, and compare it with Ru_1_/WO_2_‐800, WO_2_‐800, Ru SACs, commercial Ru/C, and Pt/C. **Figure** **3**a shows the linear sweep voltammetry (LSV) curves in 1 m KOH, where the Ru–W/WO_2_‐800 displays a remarkable onset potential near zero. A significantly reduced overpotential of 11 mV (at 10 mA cm^−2^) is confirmed for Ru–W/WO_2_‐800 (Figure S12, Supporting Information), which is lower than that for Pt/C, Ru/C, Ru_1_/WO_2_‐800, Ru SACs, and WO_2_‐800, demonstrating its superior HER performance. Moreover, Ru–W/WO_2_‐800 also reveals a low potential of 152 mV to gain a current density of 1 A cm^−2^ (Figure S13, Supporting Information). The insufficient activity of WO_2_‐800 indicates the dominance of Ru in actuating the HER process. Furthermore, the relatively higher overpotential of Ru_1_/WO_2_‐800 compared to that of Ru–W/WO_2_‐800 highlights the pivotal role of Ru—W bonding for boosting the HER pathway.

The outstanding HER activity of Ru–W/WO_2_‐800 is further demonstrated by its significantly lower Tafel slope (31.3 mV dec^−1^) compared to previously reported works (Figure 3b,c). This superior performance can be attributed to the high intrinsic HER activity of Ru–W/WO_2_‐800, which is confirmed by its notable turnover frequency (Figure S14, Supporting Information). Figure 3d shows the normalized activity based on the mass of metal, Ru–W/WO_2_‐800 exhibits a mass activity of 5863 mA mg^−1^_Ru_ at 50 mV, which is over 14 times higher than that of Pt/C (401 mA mg^−1^_Pt_), suggesting its potential for practical applications. The advantage of the synergistic Ru–W pair sites is further emphasized by the larger double‐layer capacitances (*C*_dl_) of Ru–W/WO_2_‐800 than that of Ru_1_/WO_2_‐800 (Figure 3e; Figure S15, Supporting Information), and the smaller charge transfer resistance (Figure S16, Supporting Information). The stability of Ru–W/WO_2_‐800 is assessed through various tests (Figure 3f). The results demonstrate the robustness of Ru–W/WO_2_‐800, as evidenced by the negligible increase in the overpotential after 1000 cycles and the superior long‐term stability that up to 500 h at 250 mA cm^−2^. Furthermore, the used Ru–W/WO_2_‐800 maintained its original structure, revealing good structure stability (Figure S17, Supporting Information).

Electrochemical hydrogen production exploiting seawater has drawn great attention recently, due to the huge reserves of the feedstock.^[^ ^30^, ^31^ ^]^ Thus, the hydrogen generation ability of electro‐catalysts in alkaline simulated seawater (1 m KOH and 0.5 m NaCl) is tested. As shown in Figure 3g and Figure S18 (Supporting Information), although all catalysts display a decline in the HER activity compared to that in alkaline fresh water, the Ru–W/WO_2_‐800 still exhibits a sufficient overpotential of 17 mV at 10 mA cm^−2^ and a Tafel slope of 34.1 mV dec^−1^. The robustness of the Ru–W/WO_2_‐800 in alkaline simulated seawater is further confirmed by the chronopotentiometry test conducted at 250 mA cm^−2^ for a duration of 100 h (Figure 3h). Inspired by the excellent HER performance of Ru–W/WO_2_‐800 in both alkaline freshwater and alkaline simulated seawater, the overall water‐splitting is measured, considering its significant application.^[^ ^32^, ^33^, ^34^, ^35^ ^]^ The electrolyzer is assembled by using Ru–W/WO_2_‐800 as cathode and commercial RuO_2_ as anode (Ru–W/WO_2_‐800 || RuO_2_ couple), and in reference to Pt/C || RuO_2_ couple. As depicted in Figure 3i, the Ru–W/WO_2_‐800 || RuO_2_ couple just requires 1.50 V to gain a current density of 10 mA cm^−2^ in 1 m KOH, lower than the 1.56 V for Pt/C || RuO_2_ couple. Moreover, the current density over Ru–W/WO_2_‐800 || RuO_2_ couple is much higher than that over Pt/C || RuO_2_ couple at higher voltages. The water‐splitting ability of Ru–W/WO_2_‐800 in seawater is also evaluated, and although a slight decline in activity is observed due to the obstruction of active sites by NaCl, the current density remains higher than that of Pt/C. These results highlight the application potential of Ru–W/WO_2_‐800 as cathode material for producing green hydrogen through water electrolysis.

### 2.4 Understanding the Active Sites

**Figure 4:**
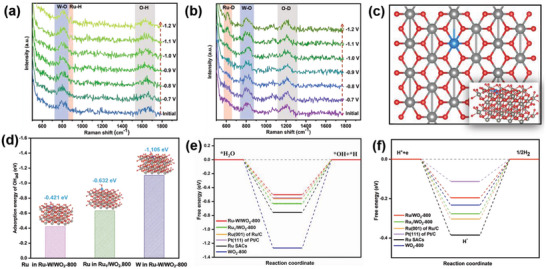
a,b) Operando Raman spectra of Ru–W/WO_2_‐800 at different potential versus Ag/AgCl in 1 m KOH in H_2_O (a) and D_2_O (b). c) Simulative structure of Ru–W/WO_2_‐800. d) Simulative OH adsorption model and corresponding adsorption energy on Ru and W sites. e) Free energy diagram for water dissociation. f) Free energy diagram for hydrogen recombination.

To elucidate the HRE process, potential dependent operando Raman spectroscopy is conducted in 1 m KOH solutions of both H_2_O and D_2_O. The operando Raman spectra of Ru–W/WO_2_‐800 are recorded at the potential from −0.7 to −1.2 V (vs Ag/AgCl), spanning the non‐Faradaic region to the HER region. **Figure** **4**a shows the Raman spectra of Ru–W/WO_2_‐800 in the 1 m KOH solution of H_2_O. The signal at 798 and 1634 cm^−1^ can be attributed to the W—O in WO_2_ and H—O—H bending mode of water, respectively^[^ ^36^, ^37^, ^38^ ^]^ Moreover, a new signal emerges at 884 cm^−1^ when the potential is shifted negatively to −1.0 (vs Ag/AgCl), and its intensity increases further upon shifting the potential to 1.2 V (vs Ag/AgCl). As reported, this signal corresponds to the Ru–H stretch.^[^ ^23^ ^]^ To verify the assignment of the Raman signal at 884 cm^−1^, 1 m KOH solution of D_2_O is used. As shown in Figure 4b, after H_2_O is changed to D_2_O, the signal at 884 cm^−1^ shifts to 614 cm^−1^, with a downward shift ratio estimated at 69.5%, which is close to the theoretical value of 71.1%, confirming the Ru–H stretch on Ru–W/WO_2_‐800. Considering the Ru–H stretch emerges at a potential as low as −1.0 (vs Ag/AgCl), efficient water dissociation is demonstrated for Ru–W/WO_2_‐800.

DFT calculations are used to further dig into the original effect of the Ru–W synergy on the HER pathway. A model featuring a bonded Ru–W pair within the WO_2_ cell is constructed based on the experimental results of Ru–W/WO_2_‐800 (Figure 4c). For comparison, the model of Ru adsorbed on the surface of WO_2_ (Ru_1_/WO_2_‐800), pure WO_2_ (WO_2_‐800), and N‐coordinated Ru single atom (Ru SACs) are also built (Figure S19, Supporting Information). The OH_ad_ poisoning effect is first investigated by evaluating the absorption energy of OH_ad_ (*E*_abs_OH). As shown in Figure 4d and Figure S20 (Supporting Information), the Ru site in Ru–W/WO_2_‐800 exhibits an E_abs_OH of −0.421 eV, higher than that of the Ru site in Ru_1_/WO_2_‐800 (−0.632 eV) and Ru site in Ru SACs (−0.632 eV), suggesting a weakening of Ru–OH interaction facilitated by the presence of the Ru–W pair. Moreover, the strong interactions of W–OH on the W site in Ru–W/WO_2_‐800 and W site in WO_2_‐800 further demonstrate the optimizing of the OH absorption over Ru–W dual atom sites in Ru–W/WO_2_‐800.

Figure S21 (Supporting Information) presents the absorption energy of H_2_O (E_abs_H_2_O), which disclosed the faster H_2_O capture on the Ru site in Ru–W/WO_2_‐800 (*E*_abs_H_2_O = −1.325 eV) compared to Ru_1_/WO_2_‐800 and Ru SACs, suggesting the acceleration of the following H_2_O dissociation step. The optimal water dissociation intermediate (H–OH) on Ru–W/WO_2_‐800 is presented in Figure S22 (Supporting Information), with the *H absorbing on the Ru site and *OH absorbing on the bonded W site. Based on this optimal structure, Ru–W/WO_2_‐800 displays the lowest H_2_O dissociation barrier of 0.50 eV compared to Ru single atom, Pt/C, and Ru/C (Figure 4e). This indicates its superior H_2_O dissociation ability. Furthermore, the optimized hydrogen recombination ability of Ru–W/WO_2_‐800 in an alkaline medium is also revealed by the DFT calculations (Figure 4f; Figure S23, Supporting Information). Overall, compared to the adsorbed Ru on WO_2_ in Ru_1_/WO_2_‐800, the bonded Ru–W pair site in the WO_2_ lattice provides Ru–W/WO_2_‐800 with solid advantages: 1) rapid hydroxy transferring performance, 2) superior H_2_O dissociation ability, and 3) optimized the hydrogen recombination. Thus, the HER performance of Ru–W/WO_2_‐800 in an alkaline medium is significantly boosted by the synergistic effect of the Ru–W pair sites.

## 3 Conclusion

In this work, we fabricate a Ru single atom doped WO_2_ by lattice confined strategy, which is identified as bonded Ru–W pairs embedding in the lattice of well‐crystallized WO_2_. The resultant Ru–W/WO_2_‐800 delivers excellent HER performance under alkaline and seawater media with ultralow overpotential and remarkable mass activity, as well as robust stability at high current density. Moreover, the Ru–W/WO_2_‐800 also presents low voltages in the overall water‐splitting system, beneficial for its practical application. The DFT calculation combining with the operando Raman spectra reveals that the outstanding HER activity stems from the synergetic effect of Ru–W pair sites via ensemble catalysis, in which the W sites expedite the rapid hydroxy transferring and water dissociation, the Ru sites accelerate the hydrogen combination process. This work may provide important inspiration for designing atomic‐scale catalysts aiming at complicated electro‐catalysis.

## Conflict of Interest

The authors declare no conflict of interest.

## Author Contributions

W.M. and X.Y. contributed equally to this work. K.L. and Y.Q. designed and wrote this work. J.B. and X.L. supervised and funded this work. W.M. conducted the catalysts synthesis and the HER evolutions. X.Y., D.L., R.X., L.N., and L.Z. gave their help in the characterizations. P.B. and S.W. designed and practiced the operando Raman measurements. Y.W., G.W., and J.D. co‐modified the paper. All authors revised the writing work of this study.

## Supporting information

Supporting Information

## Data Availability

The data that support the findings of this study are available from the corresponding author upon reasonable request.
