# Supplementary material for: Ru–W Pair Sites Enabling the Ensemble Catalysis for Efficient Hydrogen Evolution
Source: Adv Sci (Weinh). 2023 Jul 12;10(26):2303110. doi: 10.1002/advs.202303110 (PMC10502621; doi:10.1002/advs.202303110)
Supplement: Supplementary file 1 — Supporting Information [file ADVS-10-2303110-s001.pdf]

ADVANCED SCIENCE
Open Access

Supporting Information

for *Adv. Sci.*, DOI 10.1002/advs.202303110

Ru–W Pair Sites Enabling the Ensemble Catalysis for Efficient Hydrogen Evolution

Weilong Ma, Xiaoyu Yang, Dingding Li, Ruixin Xu, Liangpeng Nie, Baoping Zhang, Yi Wang, Shuang Wang, Gang Wang, Jinxiang Diao, Lirong Zheng, Jinbo Bai, Kunyue Leng, Xiaolin Li* and Yunteng Qu**

Supporting Information

Ru-W Pair Sites Enabling the Ensemble Catalysis for Efficient Hydrogen Evolution

Weilong Ma, Xiaoyu Yang, Dingding Li, Ruixin Xu, Liangpeng Nie, Baoping Zhang, Yi Wang, Shuang Wang, Gang Wang, Jinxiang Diao, Lirong Zheng, Jinbo Bai, Kunyue Leng, Xiaolin Li* and Yunteng Qu**

Materials and Methods

1.Materials:

All chemicals were analytical grade and without further purification. Analytical grade Sodium tungstate dihydrate, Ruthenium(III) chloride trihydrate, dopamine hydrochloride, Hydrogen chloride and potassium hydroxide were obtained from Innochem. Commercial Pt/C (20 wt%), Ru/C (5 wt%) and Nafion were acquired from Sigma-Aldrich.

2. Synthesis of Ru-W/WO_2-800:

Firstly, 2 mmol of dopamine hydrochloride was dissolved in 20 ml of deionized water, and the pH value was adjusted to 2 by adding 800 μl HCl (1 mol L^{-1}). Then, 0.1 mmol of $RuCl_3\cdot xH_2O$ was added to the solution and mixed by stirring to obtain a homogeneous solution. After that, 20 ml $Na_2WO_4\cdot 2H_2O$ solution (0.1 mol L^{-1}) was slowly dropped into the solution under vigorous stirring. A large amount of brown precipitate formed immediately. The reaction was further stirred for 1 h, the obtained product was collected by centrifugation, washed with deionized water and ethanol 3 times. The precursor was dried in an oven at 60 °C overnight and heated at 800 °C for 2 h in Ar atmosphere at a heating rate of 2 °C/min.

3. Synthesis of WO_2-800:

WO_2 was prepared without the addition of any other metal salts followed by the same synthetic approach of Ru-W/WO_2-800.

4. Synthesis of Ru_1/WO_2-800:

Firstly, 100 mg WO_2-800 were mixed with 50 mL ethanol and stirred for 0.5 h. After that, 2 mg $RuCl_3\cdot xH_2O$ was added in solution and further stirred for 12 h. Then, evaporated the solvent to dryness at 70 °C, and kept the obtained sample under Ar atmosphere at 350 °C for 2 h.

5. Synthesis of Ru SACs catalysts:

In a typical synthesis, 3 ml NH_4OH, 80 ml ethanol and 180 ml deionized water were mixed by stirring 10 minutes. Following, 10ml pure dopamine solution (40 mg ml^{-1}) and 10 ml Ru contained dopamine solution (DA=40 mg ml^{-1}, Ru=0.43 mg ml^{-1}) were slowly alternate dropped into the solution under vigorous stirring. The reaction was further stirred for 12 h and gradually formed brown precipitation under stirring. The obtained product was collected by centrifugation, washed with deionized water and ethanol 3 times. The precursor was dried in an oven at 60 ℃ overnight and heated at 800 ℃ for 5 h in Ar atmosphere at a heating rate of 2 ℃/min.

6.Characterization:

Scanning electron microscopy (SEM) images were obtained on a FEI Apreo S microscope. Transmission electron microscopy (TEM) and high-resolution transmission electron microscope (HR-TEM) images were collected with a Talos F200X microscope. Aberration-corrected scanning transmission electron microscopy (AC HAADF-STEM) images were collected with a JEOL JEM-ARM200F microscope. The X−ray diffraction (XRD, Bruker D8 Advance) was used to analyze crystal structures. X-ray photoelectron spectroscopy (XPS) measurements were conducted on a PHI 5000 Verasa spectrometer using Al Kα radiation. The metal contents in the catalysts were determined by Inductively Coupled Plasma Optical Emission Spectrometer (ICP-OES) on a Agilent 5110. X-ray absorption fine structure (XAFS) measurements for the Ru K-edge, W L_3-edge were performed on beamline 1W1B of Beijing Synchrotron Radiation Facility. The acquired EXAFS data were extracted and processed according to the standard procedures using the ATHENA module implemented in the IFEFFIT software packages. Operando Raman spectra were measured under controlled electrochemical potentials using a three-electrode epoxy cell with a counter electrode of Pt wire and Ag/AgCl. A controlled active area of 0.384 cm^2 by an insulation layer on carbon paper drop-casted with 0.1 mg catalyst was used as the working electrode. Raman spectra were collected using a Raman spectrometer (WITEC Alpha500) by a 633 nm He–Ne laser (Research Electro-Optics, Inc., USA) at the objective. A 20× microscope objective lens (NA=0.4, Epiplan-Neofluar, Zeiss, Germany) was used, focusing on the sample surface and avoiding the contact to the electrolyte. Acquisition time was set as 20 s for the spectral Raman shift ranging from 500 to 1800 cm^{-1} window a UHTS300 spectrometer (WITec GmbH, Germany) with a CCD camera (Andor Tech-nology, UK) operating at -60 °C. The downward shift ratio and the theoretical value was calculated based on the equation below:

$$\gamma = \frac{\nu(\mathrm{RuD})}{\nu(\mathrm{RuH})} = \frac{\sqrt{m(Ru) + m(D)}}{\sqrt{m(Ru) \times m(D)}} \div \frac{\sqrt{m(Ru) + m(H)}}{\sqrt{m(Ru) \times m(H)}}$$

Where γ represents the downward shift ratio, ν represents the Raman shift, m represents the relative molecular mass.

7. Electrochemical measurement:

All the electrochemical measurements were carried out in a conventional three-electrode cell using the CHI 760E electrochemical workstation at room temperature. A rotating disk electrode (RDE) with a glassy carbon (GC) electrode (diameter: 5 mm; area: 0.196 cm^2) was utilized as the working electrode (WE), and the Pt plate was used as the counter electrode. The Ag/AgCl reference electrode calibrated with RHE in 1 M KOH was used as a reference electrode for long-time stability measurement. For electrode preparation,10 mg of Ru-W/WO_2-800 catalyst was dispersed in the mixture solution of IPA (400 µl) and 5 wt.% Nafion (20 µl) by sonication for over 30 minutes. Then, 10 µl of the catalyst ink was drop-cast onto the surface of the GC electrode and dried in the air. Linear sweep voltammetry (LSV) plots were carried out in an Ar saturated 1.0 M KOH with a sweep rate of 1 mV s^{-1} at 1600 rpm. All of the potentials in LSV are 100% iR-corrected. The resistance for iR-compensation was tested at the open circuit potential, Ru-W/WO_2-800 (9.3 Ω), Ru_1/WO_2-800 (8.3 Ω), Pt/C (8.0 Ω), Ru/C (8.4 Ω), Ru SACs (8.9 Ω) and WO_2-800 (8.3 Ω). All the potentials were converted to the reversible hydrogen electrode (RHE) by equation.

$$V_{RHE}=E_{(VS\ Ag/AgCl)}+0.197+0.0592*pH$$

The Tafel slope was obtained the LSV curve using the equation of η=a+blogj, where a refers to the intercept, b is the Tafel slope and η denotes the overpotential. Electrochemical impedance spectroscopy (EIS) measurements were collected at η=20 mV in the frequency range from 10 kHz to 0.01 Hz, The Mass activity is calculated based on equation of Mass Activity=I/m, where I(A) is the measured current, m (mg) is the mass of Ru loaded on the GC electrode. The turnover frequency (TOF) is calculated based on the equation of TOF=I/(4Fn), where I(A) is the measured current. F is the Faraday constant (96485 C mol^{-1}). n=m/M, n (mol) is the molar amount of Ru loaded on the GC electrode, m is the mass of Ru, and M is the molecule weight. For the double-layer capacitor (C_{dl}) data, cycling voltammetry (CV) curves were recorded in the non-Faradic region with scanning rate of 5,10, 15, 20, 25 and 30 mV s^{-1}, and the C_{dl} can be obtained by plotting the current difference (Δj) against the scanning rate.

8. Computational Method:

We have employed the first-principles to perform density functional theory (DFT) calculations within the generalized gradient approximation (GGA) using the Perdew-Burke-Ernzerhof (PBE) formulation.[1-3] We have chosen the projected augmented wave (PAW) potentials to describe the ionic cores and take valence electrons into account using a plane wave basis set with a kinetic energy cutoff of 520 eV.[4,5] Partial occupancies of the Kohn−Sham orbitals were allowed using the Gaussian smearing method and a width of 0.05 eV. The electronic energy was considered self-consistent when the energy change was smaller than 10^{-5} eV. A geometry optimization was considered convergent when the

energy change was smaller than 0.05 eV $Å^{-1}$. In our structure, the U correction is used for W and Ru atoms. The Brillouin zone integration is performed using 2×2×1 Monkhorst-Pack k-point sampling for a structure. Finally, the adsorption energies (E_{ads}) were calculated as $E_{ads}= E_{ad/sub} - E_{ad} - E_{sub}$, where $E_{ad/sub}$, E_{ad}, and E_{sub} are the total energies of the optimized adsorbate/substrate system, the adsorbate in the structure, and the clean substrate, respectively. The free energy was calculated using the equation:

$$G=E_{ads}+ZPE-TS$$

where G, E_{ads}, ZPE and TS are the free energy, total energy from DFT calculations, zero-point energy and entropic contributions, respectively. The U correction for W and Ru were used as 4.18 and 4.92 eV for d orbital, respectively.

Supplementary Figures

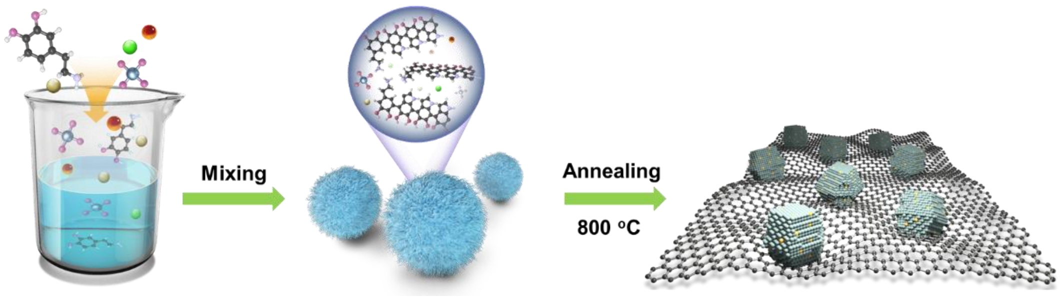

Figure S1. Illustration of the synthetic process of dual-atom Ru-W catalyst.

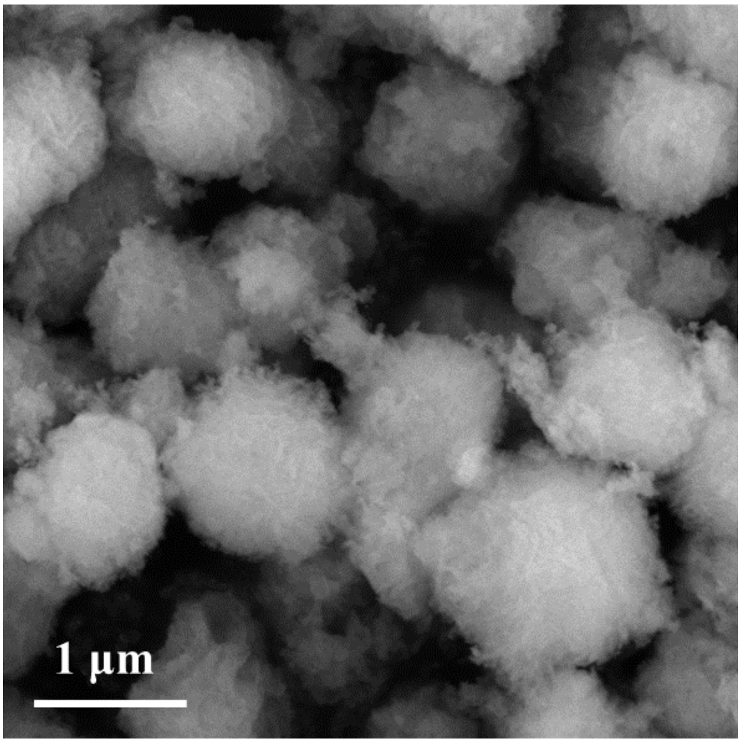

Figure S2. SEM image of as obtained mixture of polydopamine, W and Ru.

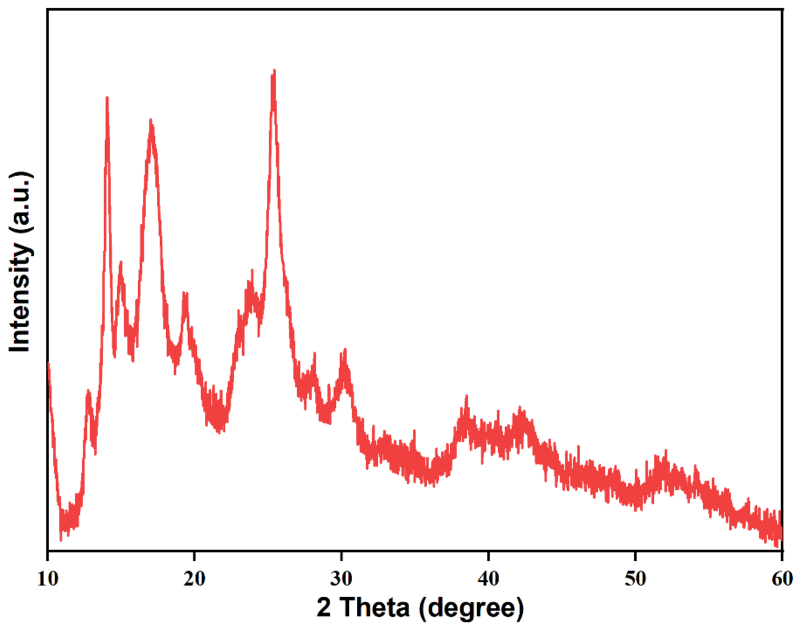

Figure S3. XRD pattern of the as obtained mixture of polydopamine, W and Ru. Only the diffraction peaks belonging to polydopamine were observed.

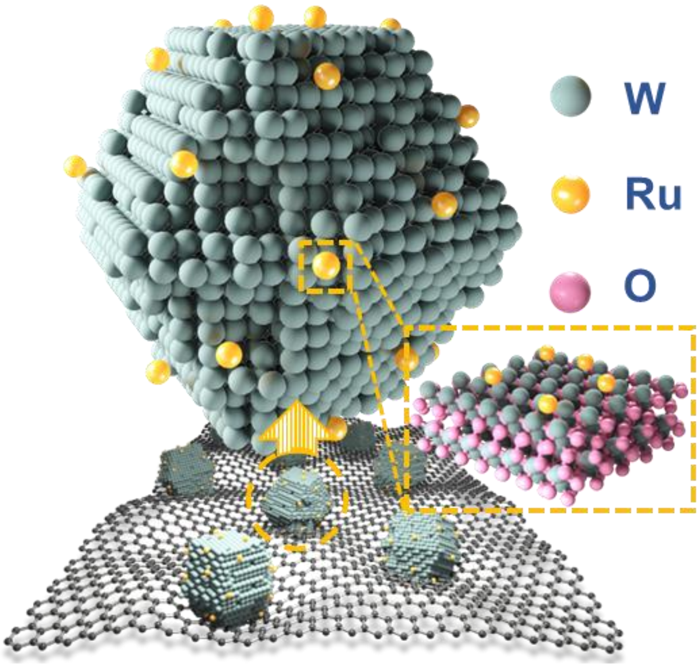

Figure S4. Simulative structure of Ru_1/WO_2-800. Single atom Ru sites randomly disperse on the surface of WO_2.

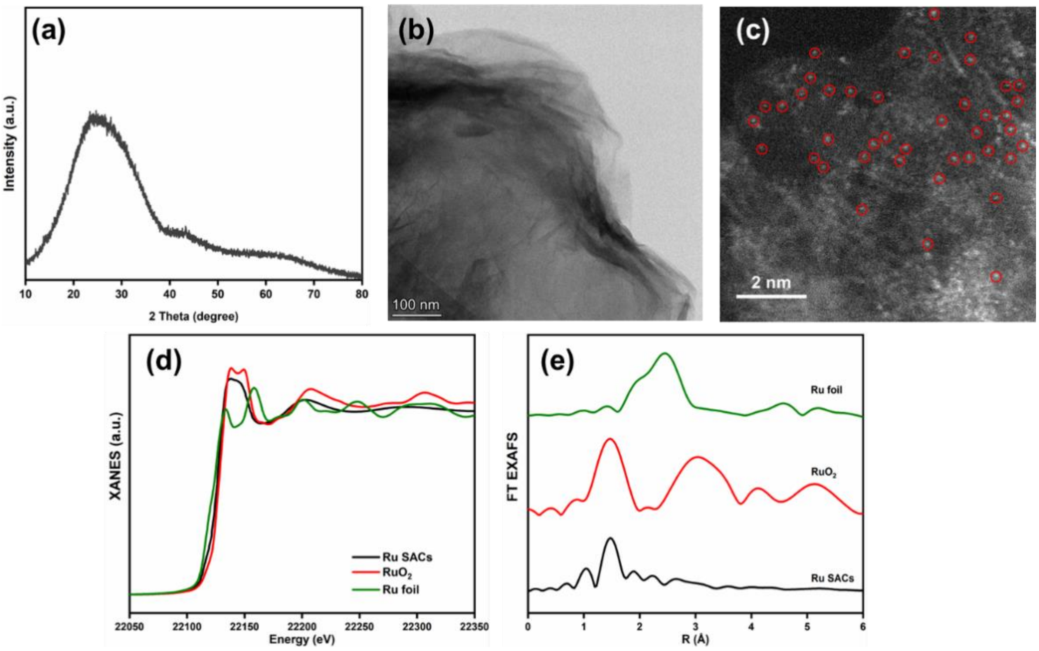

Figure S5. Structure identification of Ru SCAs. (a) XRD pattern, (b) TEM image, (c) Aberration-Corrected HAADF-STEM images, (d) Ru K edge XANES spectrum, (e) Ru K edge FT EXAFS spectrum.

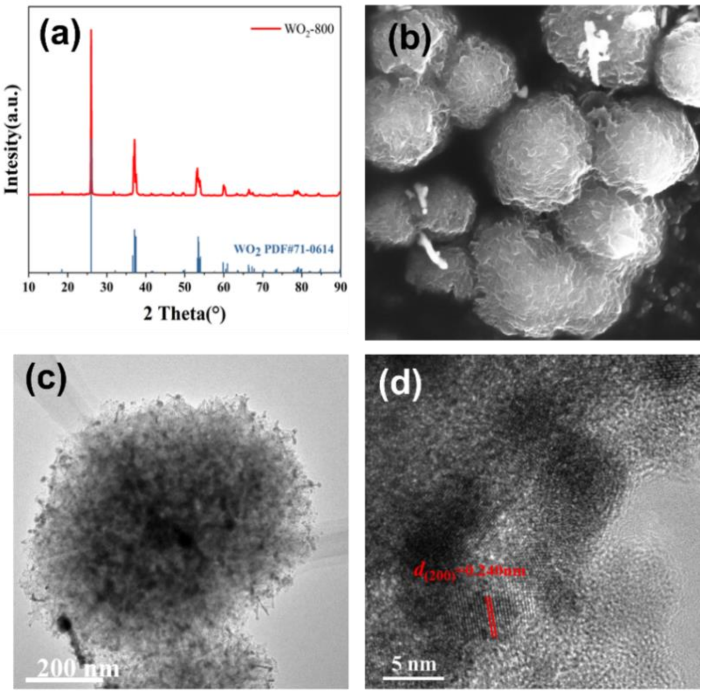

Figure S6. Characterization of WO_2-800. (a) XRD pattern, (b) SEM image, (c) TEM image, (d) HRTEM image.

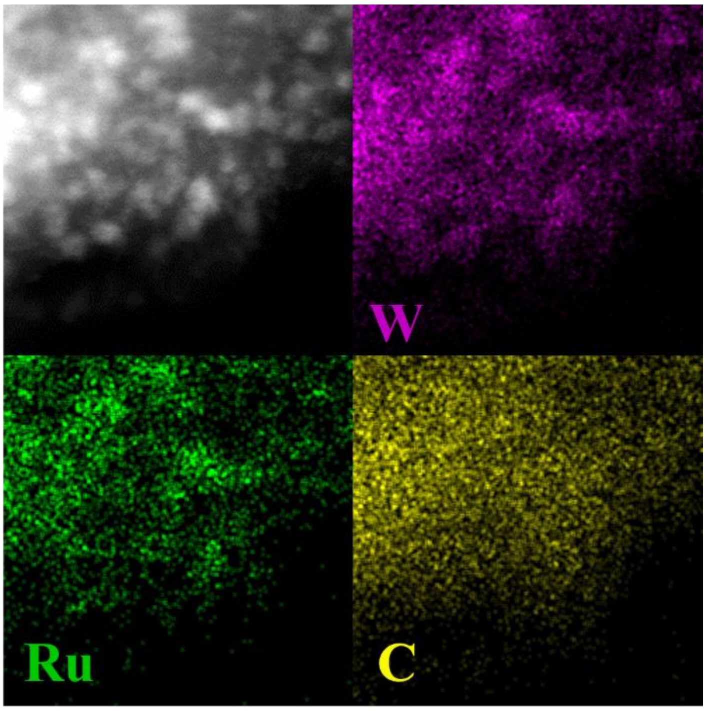

Figure S7. EDS element mapping of Ru-W/WO_2-800.

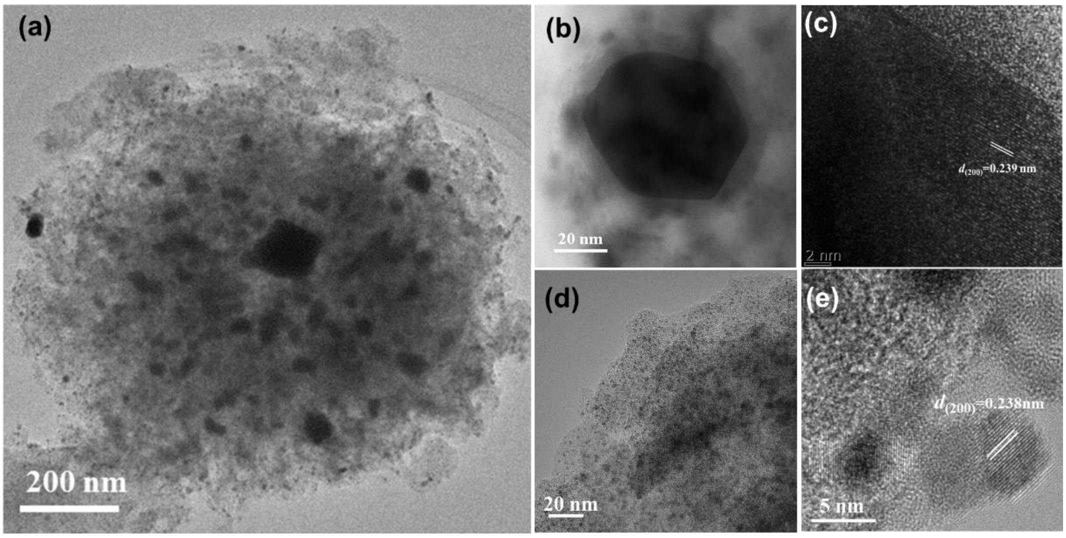

Figure S8. TEM images. (a) Ru-W/WO_2-800. (b, c) WO_2 particle in Ru/WO_2-800. (d, e) WO_2 cluster in Ru-W/WO_2-800.

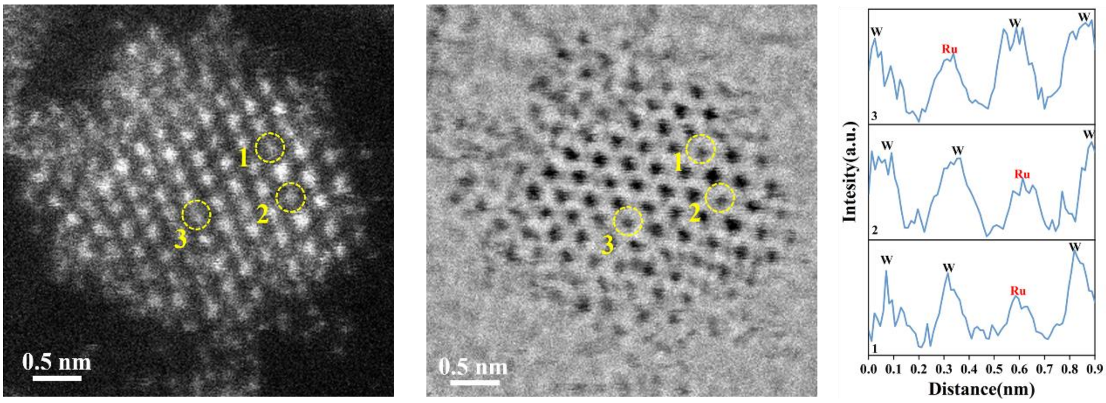

Figure S9. Dark and bright filed STME images and corresponding line intensity profiles of WO_2 clusters in Ru-W/WO_2-800.

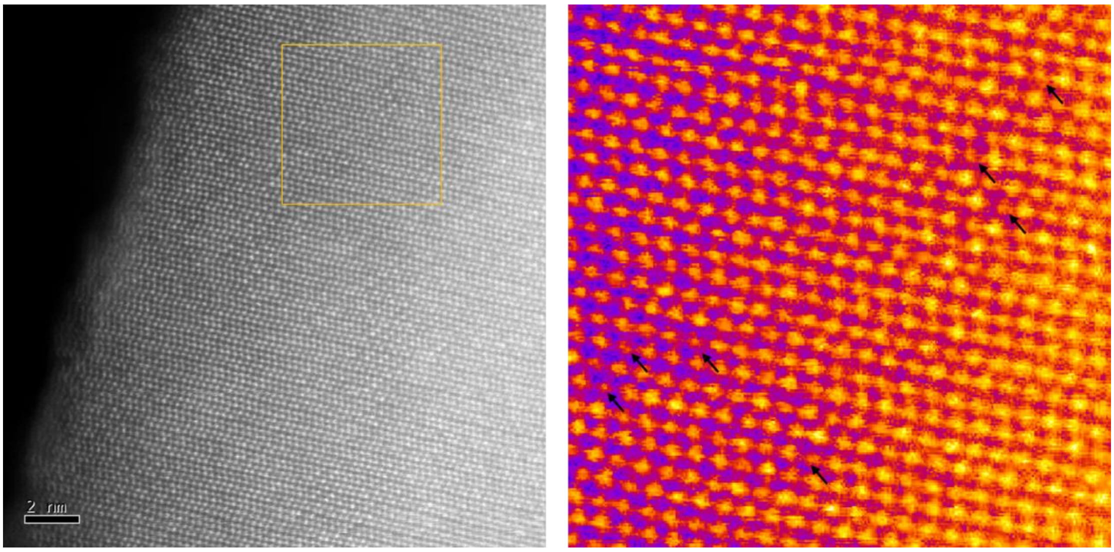

Figure S10. Aberration-Corrected HAADF-STEM images and corresponding intensity profiles of Ru_1/WO_2-800

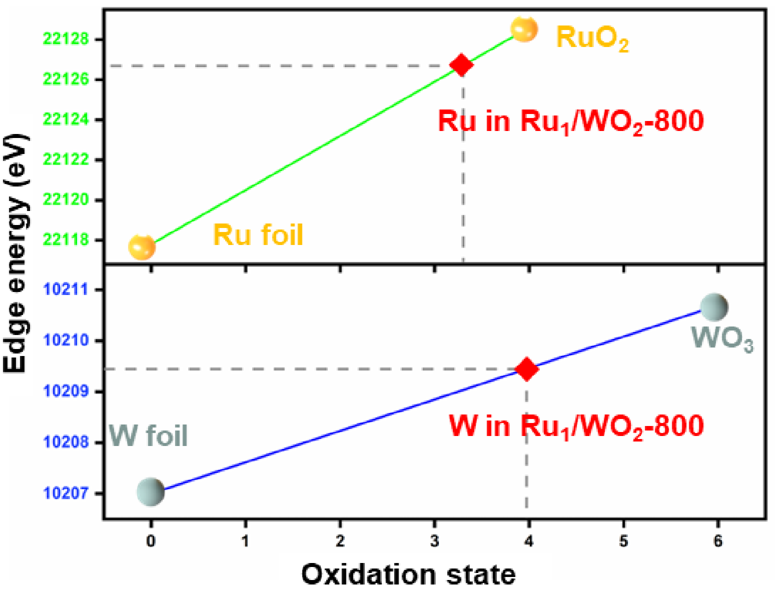

Figure S11. Simulative oxidation state of Ru and W in Ru$_1$/WO$_2$-800

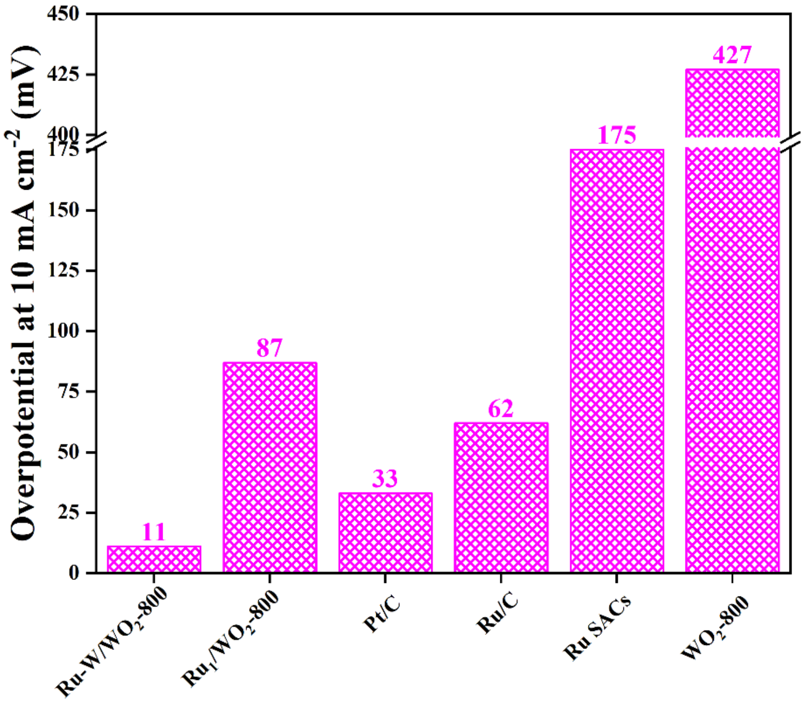

Figure S12. Overpotential of Ru-W/WO_2-800, Ru_1/WO_2-800, Pt/C, Ru/C, Ru SACs and WO_2-800 at a current density of 10 mA cm^{-2}.

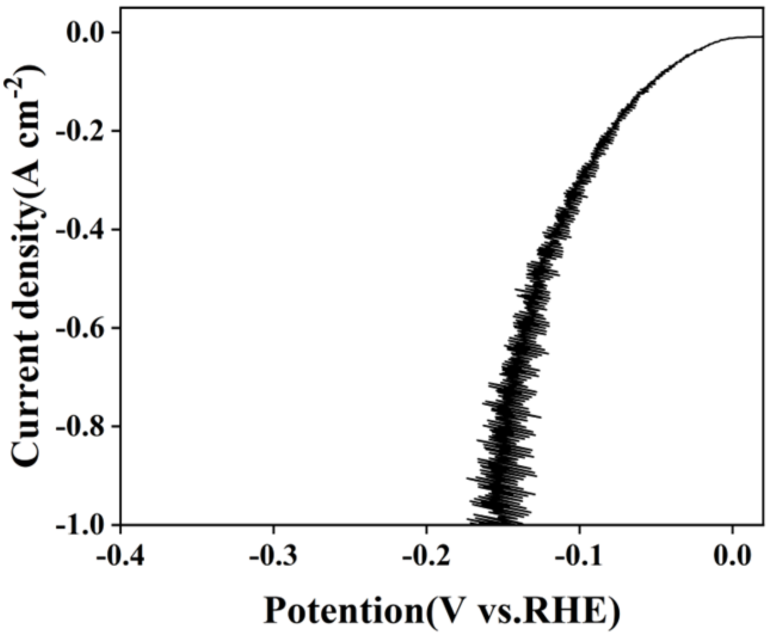

Figure S13. LSV curve of Ru-W/WO_2-800 in 1 M KOH under higher current density.

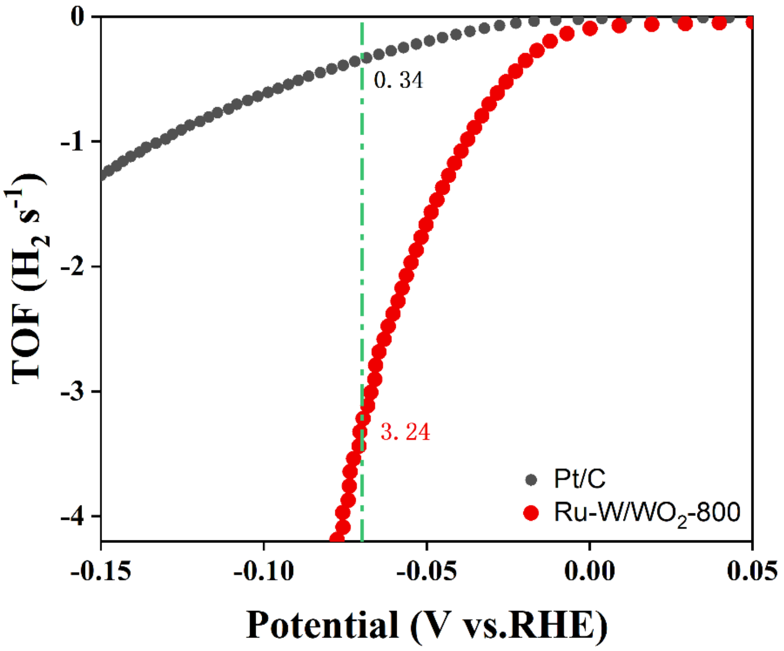

Figure S14. The turnover frequency of Ru-W/WO_2-800 and Pt/C for HRE in 1 M KOH.

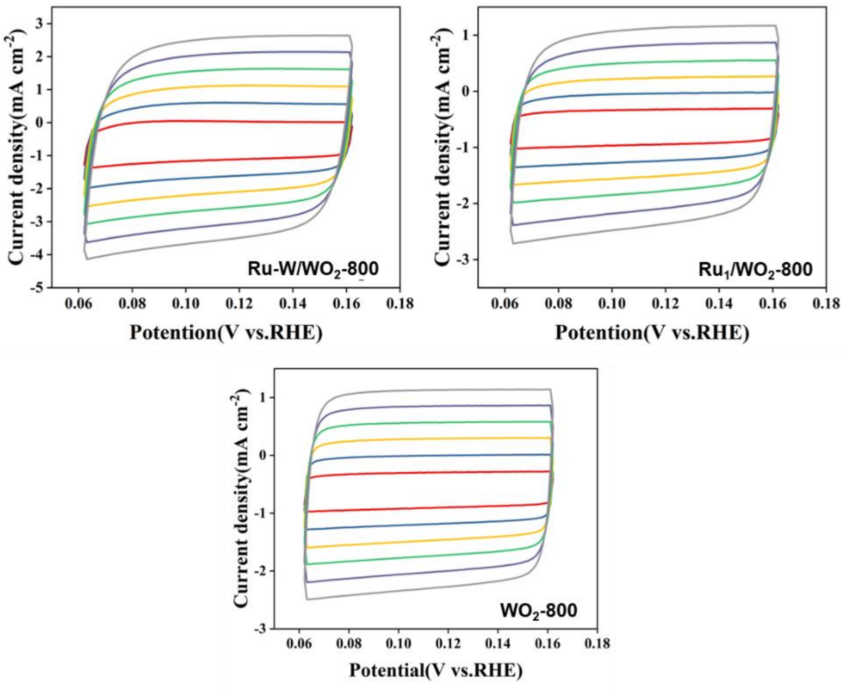

Figure S15. Cyclic voltammetry curves at different scan rates (mV s^{-1}) for Ru-W/WO_2-800, Ru_1/WO_2-800 and WO_2-800.

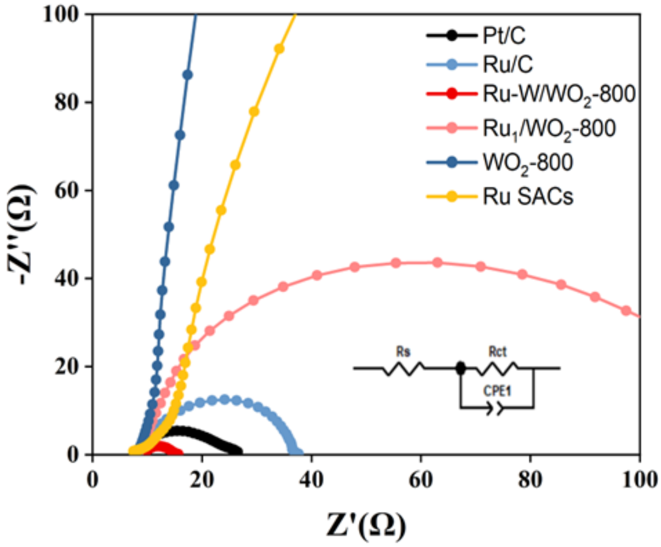

Figure S16. Nyquist plots of Ru-W/WO_2-800, Ru_1/WO_2-800, Pt/C, Ru/C, Ru SACs and WO_2-800.

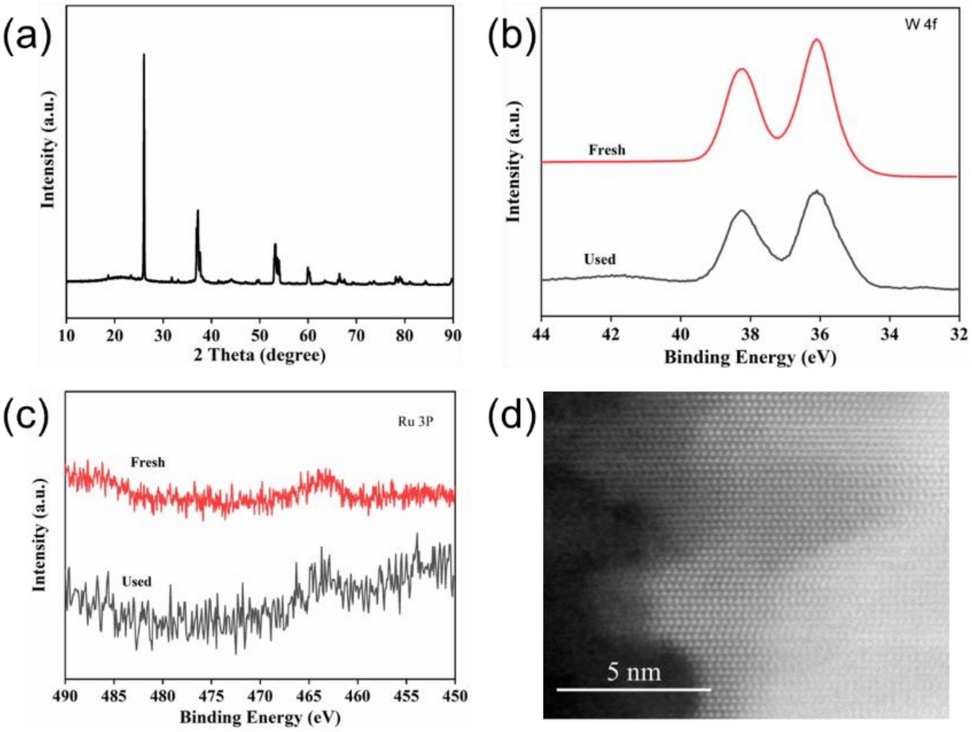

Figure S17. Physical characterization of Ru-W/WO_2 after the long-term test. (a) XRD pattern. (b) W 4f XPS spectra. (c) Ru 3p XPS spectra. (d) HAADF-STEM image.

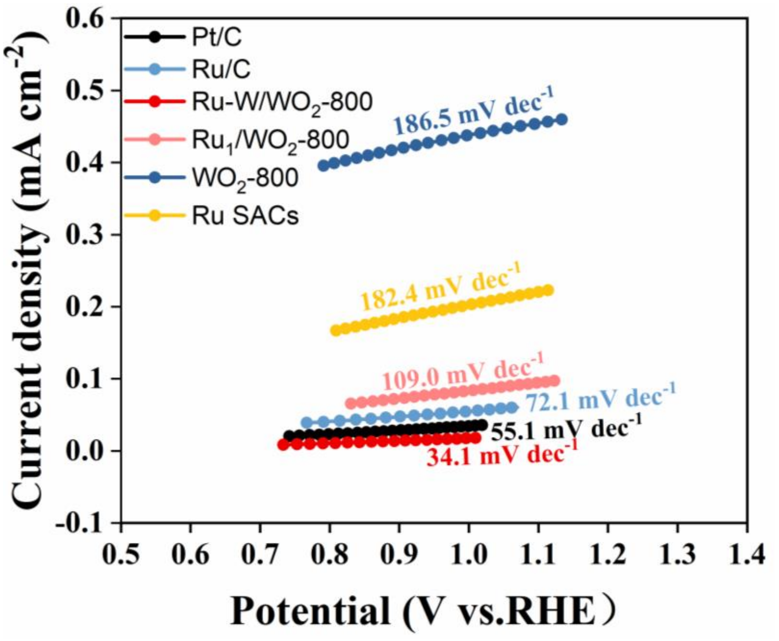

Figure S18. Tafel plots of Ru-W/WO_2-800, Ru_1/WO_2-800, Pt/C, Ru/C, Ru SACs and WO_2-800 in in alkaline simulated seawater of 1 M KOH and 0.5 M NaCl.

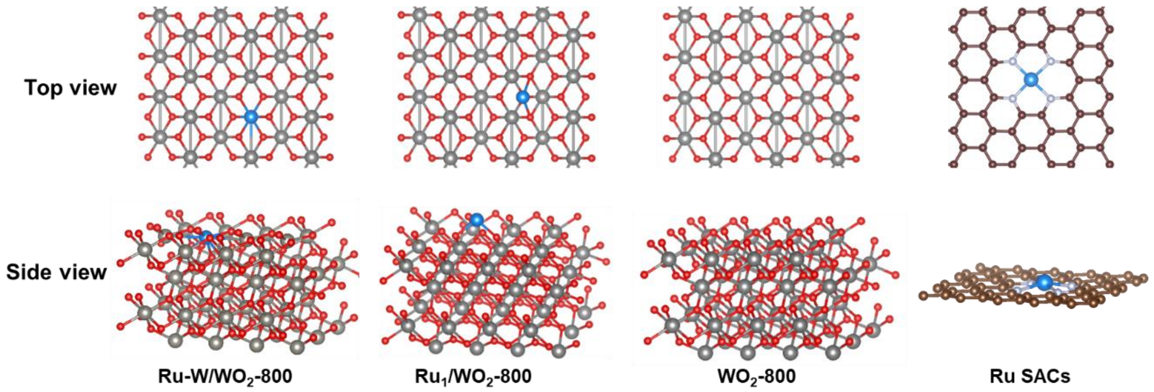

Figure S19. Simulative structures of Ru-W/WO_2-800, Ru_1/WO_2-800, WO_2-800, and Ru SACs.

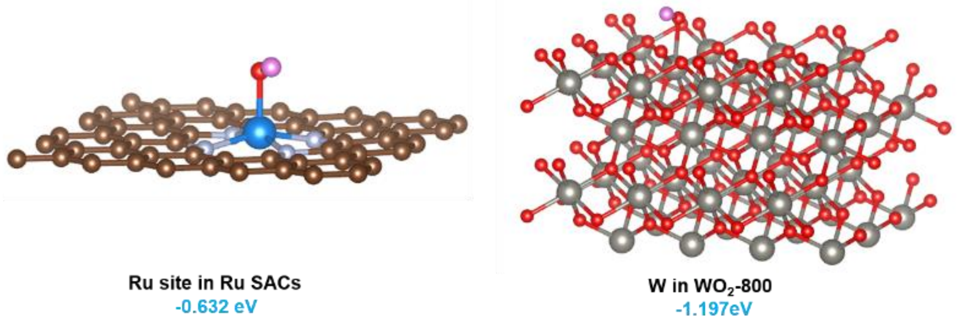

Figure S20. Simulative structure of OH adsorbed on the Ru SACs and WO_2-800 and the corresponding adsorption energy.

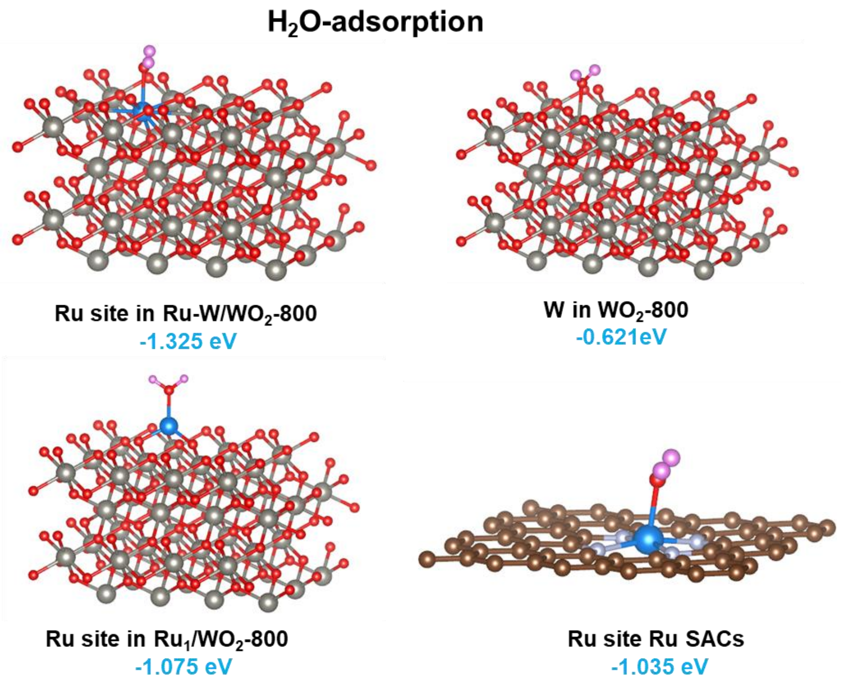

Figure S21. Simulative structures of H_2O adsorbed on Ru-W/WO_2-800, Ru_1/WO_2-800, WO_2-800 and Ru SACs and the corresponding adsorption energy.

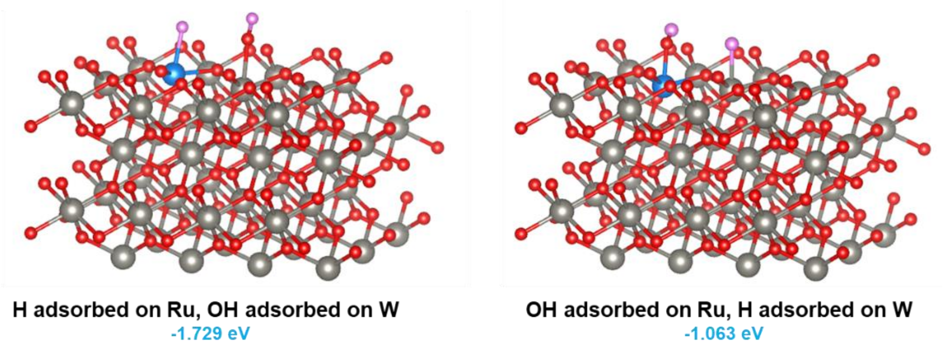

Figure S22. Different adsorption models and the corresponding adsorption energy of water dissociation intermediate (H-OH) on Ru-W/WO_2-800.

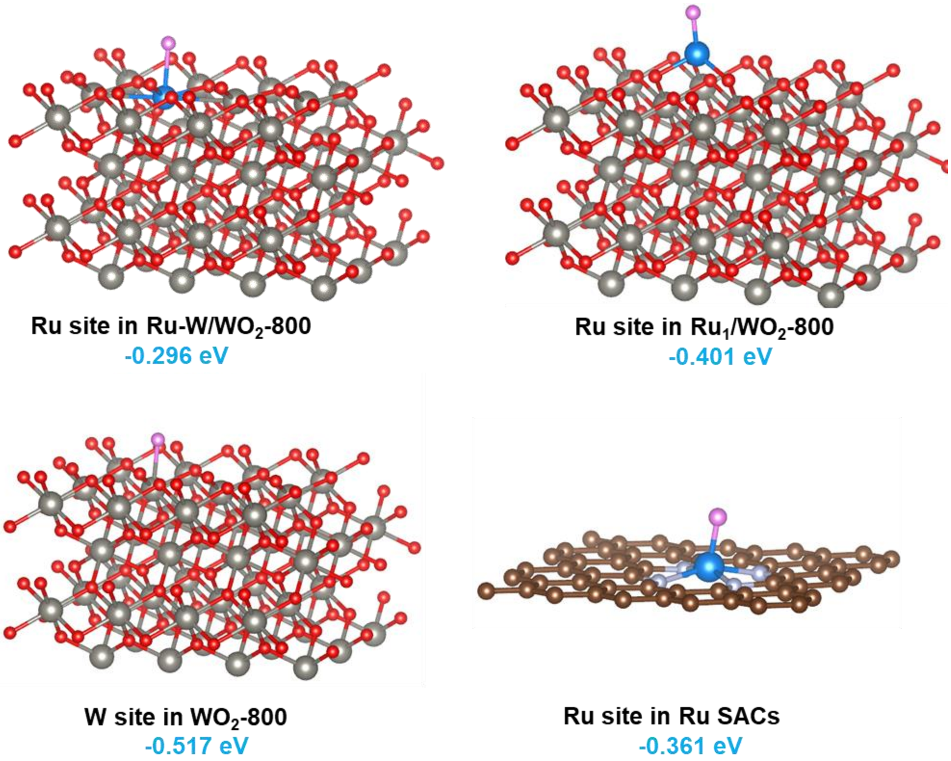

Figure S23. Simulative H adsorption model and corresponding adsorption energy on Ru and W sites

Supplementary Tables

Table S1. Metal content in various samples.

Sample	Metal content measured by ICP	
	Ru (wt%)	W (wt%)
Ru-W/WO_2-800	1.30	25.00
Ru_1/WO_2-800	1.27	25.77
Ru SACs	1.37	--

Table S2. EXAFS fitting parameters at the Ru K-edge for Ru-W/WO_2-800.

Sample	Shell	N[a]	R (Å) [b]	σ^2 (Å^2·10^{-3}) [c]	ΔE0 (eV) [d]
Ru-W/WO_2-800	Ru-O	3.1±0.2	2.01±0.03	5.2±3.0	-1.1±0.2
	Ru-W	0.9±0.1	2.79±0.02	1.9±3.0	-2.3±0.2

[a] *N*: coordination numbers; [b] *R*: bond distance; [c] σ^2: Debye-Waller factors; [d] ΔE_0: the inner potential correction.

References

[1] G. Kresse,; J. Furthmüller, Comput. Mater. Sci. 1996, 6, 15−50.

[2] G. Kresse,; J. Furthmüller, Comput. Phys. Rev. B 1996, 54, 11169−11186.

[3] J. P. Perdew, K. Burke, Phys. Rev. Lett. 1996, 77, 38655−368.

[4] G. Kresse, D. Joubert, Phys. Rev. B 1999, 59, 1758-1775.

[5] P. E. Blöchl, Phys. Rev. B 1994, 50, 17953−17979.
